# A physiological dose of oral vitamin B-12 improves hematological, biochemical-metabolic indices and peripheral nerve function in B-12 deficient Indian adolescent women

**DOI:** 10.1371/journal.pone.0223000

**Published:** 2019-10-10

**Authors:** Chittaranjan S. Yajnik, Rishikesh V. Behere, Dattatray S. Bhat, Nilam Memane, Deepa Raut, Rasika Ladkat, Pallavi C. Yajnik, Kalyanaraman Kumaran, Caroline H. D. Fall

**Affiliations:** 1 Diabetes Unit, King Edward Memorial Hospital, Pune, Maharashtra, India; 2 Epidemiology Research Unit, CSI, Holdsworth Memorial Hospital, Mysore, Karnataka, India; 3 Medical Research Council Lifecourse Epidemiology Unit, University of Southampton, Southampton, United Kingdom; University of Illinois, UNITED STATES

## Abstract

**Background:**

Vitamin B-12 deficiency is often considered synonymous with pernicious anemia, a rare condition in which severe malabsorption of the vitamin requires high-dose parenteral treatment. In developing countries such as India, inadequate dietary intake of B-12 due to socio-cultural factors leads to widely prevalent asymptomatic low B-12 status. In this scenario, lower doses of oral B-12 may be effective, safer and more affordable.

**Objective:**

To examine the effects of oral B-12 treatment at physiological doses on hematological and biochemical indices and peripheral nerve function in B-12 deficient rural Indian adolescent women.

**Methods:**

Thirty-nine women with B-12 deficiency who were excluded from a community based B-12 supplementation trial (Pune Rural Intervention in Young Adolescents (PRIYA)) received oral B-12 2μg/day, either alone (n = 19) or with multiple micronutrients (UNIMAPP formula + 20gm milk powder, n = 20) for 11 months. Hematological indices, nutrients (B-12, folate), metabolites (homocysteine) and peripheral nerve function (SUDOSCAN, Impetomedical, Paris and sensory nerve conduction velocity (NCV) of median and sural nerves) were assessed at baseline and after 11 months of B-12 treatment.

**Results:**

Results were similar in the two treatment allocation groups, which were therefore combined. At baseline, all women had B-12 concentration <100pmol/L, 79% were anemic and 33% had macrocytosis, but none had neuropathy. After 11 months of treatment, B-12 levels increased, while folate did not change. The prevalence of anemia fell to 59% and mean corpuscular volume (MCV) and plasma homocysteine concentrations decreased. Sudomotor nerve function in the feet improved by an average of 14.7%, and sensory conduction velocity in median and sural nerves increased by 16.2% and 29.4% respectively.

**Conclusion:**

We document clinically beneficial effects of supplementation with a physiological dose of oral B-12 in asymptomatic rural Indian adolescent women with very low B-12 status. These findings support a public health approach to tackle the widely prevalent low B-12 status in young Indians.

## Introduction

Vitamin B-12 deficiency is often considered synonymous with pernicious anemia, a rare condition manifesting as megaloblastic erythropoiesis, hyperhomocystinemia, methylmalonic acidemia, and neurological syndromes (subacute combined degeneration of cord, peripheral neuropathy, dementia and depression) [1]. The primary defect in this condition is a near-total block in the gastrointestinal absorption of vitamin B-12 which requires high-dose parenteral treatment. In developing countries such as India, inadequate dietary intake of B-12 due to socio-cultural factors leads to widely prevalent asymptomatic low B-12 status. In this scenario, lower doses of oral B-12 may be effective, safer and more affordable.

In the last few decades it has become increasingly apparent that inadequate dietary intake of vitamin B-12 is a widely prevalent cause of low vitamin B-12 status in many populations [2]. This predominantly affects vegetarians who do not eat adequate amounts of animal origin foods (milk, eggs, fish, chicken and meat) and lower socioeconomic groups who cannot afford them and vegans. Small doses of oral vitamin B-12 may be adequate to improve vitamin status in these individuals [3,4]. There is sparse information on this issue.

India is one such country where vitamin B-12 deficiency has been reported in substantial numbers [5–7]. This has been attributed mainly to vegetarian food habits, due to religious- socio-cultural factors and poverty, though other factors may contribute. A large majority of these individuals are asymptomatic and the B-12 deficiency may be evident only on laboratory testing. There is limited information about their clinical, hematologic and biochemical response to treatment with near Recommended Dietary Allowance (RDA) doses of vitamin B-12.

We have reported a substantial prevalence of vitamin B-12 deficiency in people living in and around the Indian city of Pune [8]. The majority are asymptomatic but have demonstrable derangements in hematological and biochemical parameters [8]. Low maternal B-12 status is prevalent in pregnancy and is associated with fetal growth restriction [9,10] and with insulin resistance in the offspring [11,12]. These findings suggested a need for B-12 supplementation using a public health approach. In preparation for a large supplementation trial we demonstrated adequate absorption of vitamin B-12 in the majority of this population using a modified CobaSorb test (oral 2μg B-12 x 3 doses) [13] and also documented improved B-12 status with near RDA (“physiological” 2 μg/day) doses of oral vitamin B-12 over one year [14]. The PRIYA trial (Pune Rural Intervention in Young Adolescents, ISRCTN 32921044) [15], was thus designed to improve the vitamin B-12 status of adolescent participants in the Pune Maternal Nutrition Study (a preconceptional birth cohort established in 1994–96) with an aim to improve health outcomes in their offspring (Intervention started in 2012).

PRIYA is a placebo- controlled trial and therefore we decided to exclude and treat those with very low vitamin B-12 status (<100 pmol/L) for ethical reasons. The current study was designed to document the effects of treatment in this excluded group. We performed two sets of measurements to document the effect of an oral physiological dose of B-12 (2μg/day), with the following objectives: 1) To document changes in clinical, hematological, biochemical and nerve function. 2) To study 1-Carbon metabolic cycles after a methionine load using stable isotopes. The first objective would provide useful information to guide public health policy about B-12 supplementation among women in the reproductive age group. And the second objective would provide mechanistic information in 1-carbon cycles to improve scientific understanding. The results of the isotopic metabolic studies have been published [16]. We now report the clinical, hematological, nutritional, biochemical-metabolic and peripheral nerve function measurements. We hypothesised that treatment with oral physiological dose of 2 μg/day B-12 for 11 months would improve the above parameters favorably in these women.

## Materials and methods

Before starting the PRIYA trial, we measured plasma B-12 concentrations in all adolescents in the Pune Maternal Nutrition Study (PMNS) cohort. We decided to exclude those with B-12 concentrations below 100 pmol/L (15th centile) from the trial and to treat them with B-12 because it would be ethically inappropriate to randomize them into a placebo-controlled trial. According to Indian nutritional guidelines (Indian Council of Medical Research, National Institute of Nutrition) the recommended daily dietary intake of B-12 is 1 μg/day[17]. This study was approved by the Institutional Ethics Committee of KEM Hospital Research Center, Pune. Approval No. 1112A(i). The ethics committee of the KEM Hospital Research Centre approved a treatment dose of 2 μg B-12 on the basis of our previous demonstration that this dose was adequately absorbed by the majority in this population, and that it was also efficacious in improving B-12 status in a one year pilot trial[18]. Informed consent was obtained from the parents and assent from the participants who were younger than 18 years of age. The study was carried out in 2012–2014.

Participants were allocated to receive either (a) vitamin B-12 only (cyanocobalamin 2μg/day) or (b) cyanocobalamin 2 μg/day + a multiple micronutrient tablet (UNIMAPP formula without folic acid) + 20 gm milk powder made up with water or added to food to mimic the two active treatment arms in the PRIYA trial[15]. All participants received 100 mg iron and 500 μg folic acid tablet once a week as per Government of India guidelines.

Measurements were carried out at baseline and after 11 months of treatment. Physical examination included anthropometry (height and weight) and examination for stomatitis, glossitis and peripheral neuropathy (clinical examination for joint sense; vibration, using a 128 Hz tuning fork; and light touch, using a Semmes Weinstein monofilament 5g, classified as normal, impaired or absent sensation). Blood samples were centrifuged in cold at 4°C within one hour of sample collection and the separated plasma and serum were stored at -80°C for analysis later. Hematological measurements (Complete Blood Count) were done on a Beckman Coulter Ac·T diff Analyzer (Miami, Florida, USA). Plasma vitamin B-12 was measured by microbial assay using a colistin sulphate-resistant strain of L. leichmanii.[19]. Plasma folate was measured by microbiological assay using a chloramphenicol-resistant strain of L. casei.[20] Plasma total homocysteine (tHcy) concentration was measured by reducing oxidized thiols with sodium borohydride followed by conjugation of thiols with monobromobimane [21]. The baseline and post treatment samples were processed separately, within 3 months of sample collection. Inter—and intra-batch coefficients of variation for B-12 folate and homocysteine measurements were all < 8%.

We used SUDOSCAN (Impeto Medical, Paris) which is a noninvasive and quick method for assessing dysfunction in thin unmyelinated sympathetic C nerve fibers supplying the sweat glands. We have previously demonstrated its usefulness in the diagnosis of peripheral neuropathy in diabetic patients [22,23]. Higher ESC values (Electrochemical Skin Conductance = current/power in microSiemens) indicate better nerve function and an ESC value of < 40μS is taken as diagnostic of clinically significant neuropathy (as provided by the manufacturer). Lower limbs are more sensitive to nerve tissue function changes, and hence we measured the ESC values in the feet in this study. Nerve conduction studies were performed antidromically to measure sensory nerve conduction velocities in both sural nerves of lower limb and both median nerves of upper limb (Nicolet Biomedical EMG/NCS system).

### Statistical analysis

The outcomes included plasma vitamin B-12 and tHcy concentrations, hemoglobin, mean corpuscular volume (MCV) and red cell diameter (RDW). Additional outcomes were clinical peripheral neuropathy measures, SUDOSCAN ESC measurements, and sensory NCV values of the median and sural nerves; all nerve measurements for the right and left sides were averaged for the analysis. Many variables were not normally distributed and hence non-parametric statistical tests were applied. Data are presented as median and 25^th^ & 75^th^ percentile values. The change in measurements from baseline to follow up were tested using the Wilcoxon paired signed rank test.

## Results

Of the 690 adolescents screened for the PRIYA trial, 117 (52 women) were found to have a plasma vitamin B-12 concentration <100 pmol/L. Of the 52 women, 39 agreed to research measurements and were allocated to receive either (a) vitamin B-12 only [n = 19] or (b) Vitamin B-12 + a multiple micronutrient tablet + milk powder [n = 20].

Measurements were available on 39 women participants at baseline and for 34 women at the 11 month (post-treatment) follow up. Of the five women not studied at follow up, three were pregnant and two declined follow up measurements. These five women had comparable baseline plasma vitamin B-12 concentration (median 93.4 vs 93.0 pmol/L) and SUDOSCAN ESC values (75.9 vs 76.0 μS) compared to the rest of the study group. The baseline and post treatment values of the outcomes were similar in the two treatment allocation groups (B-12 vs B-12 + MMN) and hence pooled results are presented (**Table 1).**

**Table 1:** Comparison of clinical, hematological and nerve function measurements before and after 11 months of treatment.

	Pre—Intervention		Post—Intervention		p–value
	N	Median (P25, P75)	N	Median (P25, P75)	
Height (cms)	39	155.9 (152.9, 161.1)	34	156.0 (152.7, 161.5)	<0.001
Weight (kg)	39	46.2 (41.0, 52.5)	34	46.9 (42, 53.4)	0.028
BMI (kg/m2)	39	18.6 (17.2, 21.2)	34	18.8 (17.5, 21.6)	0.248
Hemoglobin (gm%)	39	11.3 (10.5, 11.9)	34	11.8 (11.4, 12.5)	<0.001
MCV (fl)	39	94.2 (85.2, 100.8)	34	85.4 (82.4, 91.1)	<0.001
RDW (%)	39	15.1 (14.1, 16.4)	34	13.6 (12.9, 14.5)	0.001
VitaminB-12 (pmol/L) (Normal > 150pmol/L)	39	92.0 (78.0, 106.0)	34	161.5 (125.5, 226.3)	<0.001
Folate (nmol/L) (Normal > 7nmol/L)	38	18.9 (13.1, 25.8)	30	19.7 (13.5, 26)	0.795
Homocysteine (μmol/L) (Normal < 15 μmol/L)	39	41.5 (26.7, 56.7)	34	13.3 (9.7, 24.6)	< 0.001
ESC values in the feet (μS)	39	76.0 [67.7, 83.3]	33	88.5 (77.4, 92.5)	< 0.001
Median Nerve– Sensory NCV (m/s)	31	54.3 (50.7, 58.8)	26	62.3 (55.7, 68.6)	< 0.001
Sural Nerve– Sensory NCV (m/s)	31	46.4 (43.9, 52.0)	27	61.1 (54.1, 65.9)	< 0.001

p–values were calculated using the Wilcoxon paired Signed Rank test

As a group, these women were ‘undernourished’ (18 women had a body mass index (BMI) <18.5 kg/m^2^), 31 (79%) had anemia (Hb <12 g/dl) and 13 (33%) had macrocytosis (MCV >100 fl). All had normal plasma folate concentrations but high homocysteine. None of the women showed clinical signs of B-12 deficiency (stomatitis, glossitis, peripheral neuropathy) and the SUDOSCAN ESC values and sensory NCVs were in the normal range (Table 1).

After 11 months of treatment, there was a significant increase in plasma vitamin B-12 concentrations; only 4 still had a B-12 level <100 pmol/L. The hemoglobin concentration increased significantly, while the MCV, RDW, and prevalence of anemia (59%) decreased. Of the 13 women who were macrocytic at baseline, two did not attend for follow up, and of the remaining 11, the MCV had fallen to <100fl in eight. There was a substantial fall in plasma tHcy concentration, while folate concentrations remained stable. Joint position and vibration sense remained normal in all but there was a significant increase in the SUDOSCAN ESC and the median and sural sensory NCV (by 14.7%, 16.2% and 29.4% respectively).

## Discussion

In this study of asymptomatic vitamin B-12 deficient adolescent women we observed a significant improvement in B-12 concentrations, hematological and biochemical, metabolic parameters and peripheral nerve function after 11 months of treatment with an oral B-12 dose of 2 μg /day. This was not a clinical trial, but rather a care protocol, in which we documented the effects of a physiological dose of B-12 on different parameters in otherwise asymptomatic but clearly B-12 deficient women. We piggy-backed these observations onto a randomized controlled trial of B-12, in adolescents by following the B12 deficient women who were excluded from the main trial due to the ethical imperative of a placebo controlled trial. The selection of the dose of B-12 (2 μg/day) was based on two previous studies in this population in which we had demonstrated adequate absorption of oral B-12 in the majority, and documented improvement in B-12 status after oral supplementation for 1 year. The improvements in hemoglobin, MCV and RDW, homocysteine and peripheral nerve function (even though this was in the clinically normal range at baseline) are very encouraging. The substantial prevalence of vitamin B-12 deficiency in this population is at least partly ascribable to vegetarianism due to religious and socioeconomic factors. The lack of clinical symptoms (severe anemia, stomatitis and glossitis, and peripheral neuropathy) is a surprising but common observation in our experience. We do not know the reasons for this with confidence, but it could be partly due to an adaptation (in a population likely to have been B-12 deficient for many generations) or due to adequate status of the related vitamin folate which might compensate for some of the actions of vitamin B-12. These facts supported our decision to use oral B-12 in physiological doses in the PRIYA trial as well as in the treatment of excluded women. In the only other community-based intervention in adolescent Indian women, oral vitamin B-12 at a much higher dose (500 μg/day for the first 6 weeks and 15 μg/day for the subsequent 20 weeks) along with Iron-Folic acid supplementation resulted in a reduction in vitamin B-12 deficiency from 63.3% at baseline to 40.4% post intervention [24]. Such large doses are costly, and are unlikely to be used in a large-scale public health intervention in India. Other studies from India have been hospital-based and in symptomatic vitamin B-12 deficient patients. They reported an improvement in nerve conduction [25,26], cerebral blood flow (functional magnetic resonance imaging) and white matter microstructural integrity (Diffusion Tensor Imaging) [27–30] after 6–12 months of treatment with large doses of parenteral B-12.

The strengths of this study are that we measured the effects of a ‘pragmatic’ physiological dose of B-12 and demonstrated significant improvement in several important physiological parameters, making the findings highly policy-relevant. Limitations include: 1) We did not formally assess compliance with treatment, 2) small sample size because the study was piggybacked onto an ongoing trial 3) absence of a control group reflects the ethical imperative of not withholding treatment in a more severely deficient 4) lack of active B-12 and methyl malonic acid (MMA) measurements, (specific metabolic markers for B-12 deficiency), due to limited availability of funds. However, in the absence of concomitant changes in folate status, homocysteine concentrations provide important information on tissue (liver) effects of B12 supplementation, as demonstrated in our previous observational and interventional studies[18].

## Conclusion

In this community-based study in adolescent rural Indian women with asymptomatic vitamin B-12 deficiency we were able to demonstrate improvement in hematological and biochemical-metabolic indices, and in peripheral nerve function, after treatment with a physiological dose of oral vitamin B-12. Our findings are relevant to public health because of the widely prevalent low B-12 status in Indians and the practical applicability of our approach. The study provides scientific evidence to support oral B-12 supplementation in young adolescent Indians as a public health measure.

## Supporting information

S1 Table Data used for analysis in the manuscript. (XLSX)
